# Robust Biomarker Screening Using Spares Learning Approach for Liver Cancer Prognosis

**DOI:** 10.3389/fbioe.2020.00241

**Published:** 2020-04-03

**Authors:** Aman Chandra Kaushik, Aamir Mehmood, Dong-Qing Wei, Xiaofeng Dai

**Affiliations:** ^1^Wuxi School of Medicine, Jiangnan University, Wuxi, China; ^2^School of Life Sciences and Biotechnology, Shanghai Jiao Tong University, Shanghai, China; ^3^Peng Cheng Laboratory, Shenzhen, China

**Keywords:** biomarkers, spares learning, liver cancer, prognosis, ncRNA

## Abstract

LncRNAs, miRNAs, mRNAs, methylation, and proteins exert profound biological functions and are widely applied as prognostic features in liver cancer. This study aims to identify prognostic biomarkers’ signature for liver cancer. Samples with inadequate tumor purity were filtered out and the expression data from different resources were retrieved. The Spares learning approach was applied to select lncRNAs, miRNAs, mRNAs, methylation, and proteins’ features based on their differentially expressed groups. The LASSO boosting technique was employed for the predictive model construction. A total of 200 lncRNAs, 200 miRNAs, 371 mRNAs, 371 methylations, and 184 proteins were observed to be differentially expressed. Five lncRNAs, 11 miRNAs, 30 mRNAs, 4 methylations, and 3 proteins were selected for further evaluation using the feature elimination technique. The highest accuracy of 89.32% is achieved as a result of training and learning by Spares learning methodology. Final outcomes revealed that 5 lncRNA, 11 miRNA, 30 mRNA, 4 methylation, and 3 protein signatures could be potential biomarkers for the prognosis of liver cancer patients.

## Graphical Abstract

The graphical abstract depicts the pipeline of methodology that represents the flow of work for identification of prognostic biomarkers for liver cancer.

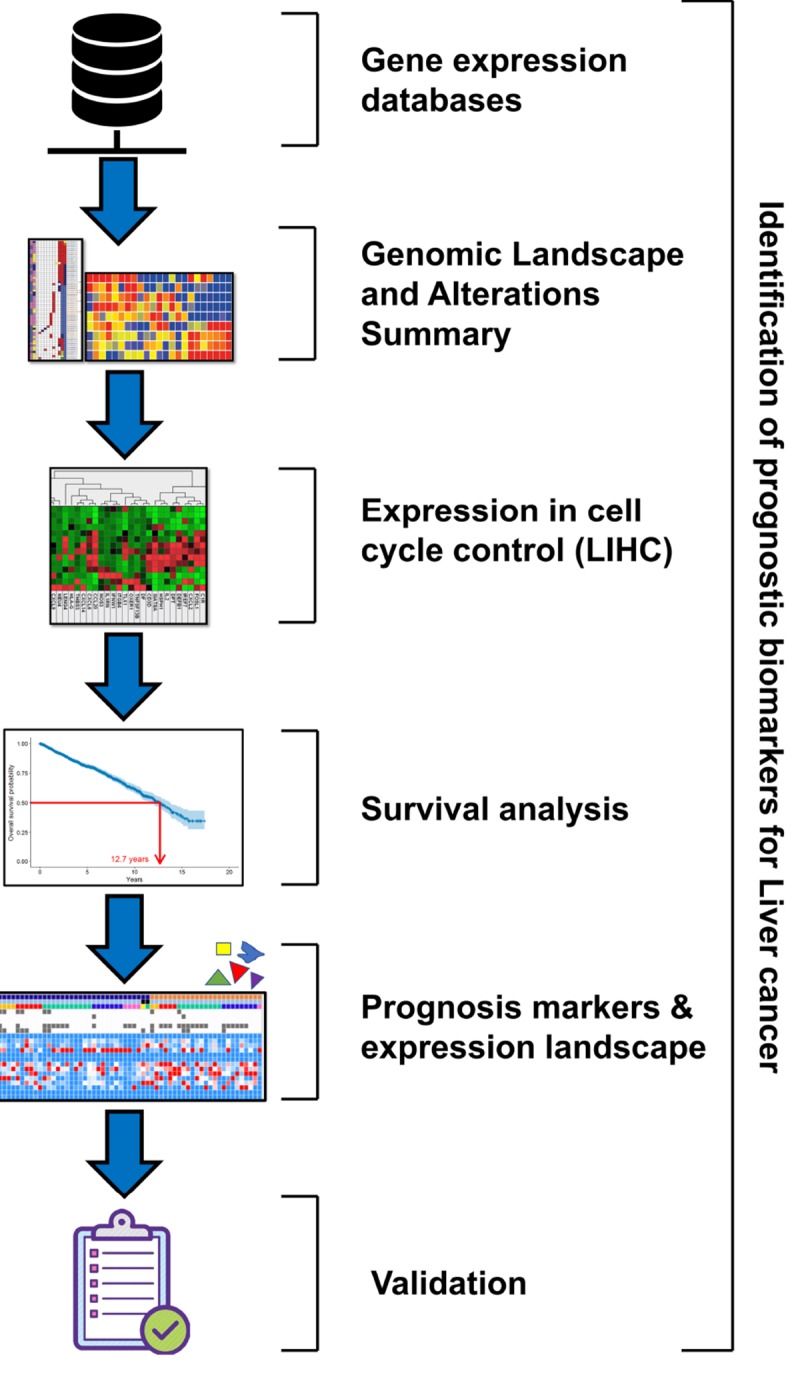

## Introduction

One of the largest organs in the human body is the liver, which is crucial for metabolism and is helpful in detoxification and maintaining homeostasis. Many ailments are concerned with the liver including hepatitis, fibrosis, genetic and metabolic issues, and liver cancer, which is one of the leading causes of cancer-related expiries (Bray et al., 2018; Dooley et al., 2018; Yang et al., 2019). Hepatocellular carcinoma (HCC) can occur in an ailing liver and encompasses numerous molecular cascades (Kanda et al., 2019). It is reported that more than 90% of liver cancers are HCC, which is an extremely assorted form of cancer verified by high-throughput sequencing and gene expression profiling, at both the molecular and histological levels (Calderaro et al., 2019).

Gene therapy has progressed as an effective source of dealing with disease-causing gene imperfections to attain a typical status. The approaches employed to treat illness by gene therapy consist of gene replacement, gene restoration, gene extension, gene muzzling, vaccination, and, currently, gene-editing technology (Alsaggar and Liu, 2016; Chew et al., 2016; Karimian et al., 2019). Thus, the identification of a gene that can be used as a potential biomarker is an important step in the treatment of liver cancer (West et al., 2019; Zheng et al., 2019; Lu et al., 2020; von Felden and Villanueva, 2020).

Statistical approaches like artificial neural networks employing BI-RADS (Baker et al., 1995) and logistic regressions have been used in several reports to improve diagnostic performance. It is most beneficial to use statistical approaches as they enhance the identification of breast cancer, with BI-RADS, as well as along the medical and statistical information concerning infected persons’ statistical threat aspects (Chhatwal et al., 2009). Regression processes go through overfitting once the prognostic covariates are involved in a large number. Similar situations lead to the well-fitting of a deterioration mockup into the drilling information. However, this doesn’t go parallel with the cases of the real world. Variable selection becomes a necessity in an attempt to get exact predictions associated with covariates of a large number, for instance, BI-RADS qualifiers and statistical data. A very famous fact claims the unfavorability of regular step-by-step assortment methods in case of regression models that have numerous covariates (Houssami et al., 2004). On the other hand, sparse penalized methods, like the minimum complete reduction and assortment operative (LASSO), together have gained ample consideration. LASSO is a penalized regression technique that approximates the deterioration constants through enhancing the log-similarity purpose (or adding the squared remainders) having restriction that the addition of the total scores of the deterioration constants, Σkj = 1||βj||, is </= to a positive constant *s*. LASSO has one of the most fascinating characters that the approximated value belonging to deterioration constants is tenuous in nature, indicating a lot of components that are accurately 0. This proves that unnecessary covariates are automatically deleted by LASSO. It is believed that LASSO has numerous required characteristics that are compulsory for the deterioration mockups with a huge covariate count. Optimization algorithms for the rectilinear deterioration model as well as for general rectilinear mockups are available in large numbers, with good efficiency. As per our information, this work is the first attempt to build a calculated LASSO deterioration mockup that could assist in the diagnosis of breast cancer based on statistical and radiological findings.

This study is aimed to compare the productivity of graphical examination to forecast liver cancer dependent on whether the calculated LASSO deterioration or bit-by-bit calculated (SL) deterioration was employed, along with evaluating the practicality of integrating statistical data into the graphic breakdown for the sake of improving liver cancer diagnosis.

## Materials and Methods

### Gene Expression Databases

The Cancer Genome Atlas (TCGA)^1^ catalog can be accessed to gain information regarding alterations in the gene, long non-coding RNAs (lncRNAs), methylations, miRNAs, mRNAs, CAN, mutational expression, and proteins involved in HCC. It is a freely accessible repository at the TCGA (Cerami et al., 2012; Gao et al., 2013). The cancer study “HCC were obtained from TCGA” and information type precedence “Mutation and CNA (DNA copy-number alterations)” were selected before analyzing genomic alterations of cell cycle control in the TCGA data on HCC. This did not require any statement of approval or informed consent for the reason that the information is retrieved from a public repository.

### Genomic Alterations Summary

Genomic modifications of cell cycle control via tumor samples were summarized. Genomic modifications inclusive of mutations, CNA (amplifications and homozygous deletions), glyphs, and dye tagging were practiced to summarize the gene expression variations. It was the first step to understand various types of gene signaling in HCC. The shared exclusivity and co-occurrence among cell cycle control were studied as well. Discordant, gene-related happenings linked with a specific cancer are most of the time conflicting in a cluster of tumors, i.e., only single biological happening is expected to occur in every sample of cancer. Another condition is the simultaneous incidence that several genes are changed in each sample (Gao et al., 2013); this was an introductory way to collect information related to various gene signaling in HCC.

### Mutations in Cell Cycle Control in HCC

Through the mutations of cell cycle control, the rate and position for all mutations within Pfam protein fields were specified. Colored bars denote an entire extent of cell cycle control proteins and the base of every bar in gray denotes the amino acid count. Protein domains are represented by the boxes colored in red, blue, and green. The lines and points signify the position and rate of genes. The frameshift or nonsense mutations are shown in red, missense mutations are in green, and the black color represents the in-frame deletions (Fang et al., 2015).

### Survival Analysis

The survival analysis bears great importance in prognosis to highlight changes in the survival rate. Here, the differences in the overall survival were evaluated via survival analysis among samples having a single or more alteration as that of the inquired genes(s) and also the samples that have no variation.

### Statistics

To carry out correlation analysis, a scattered graph of lncRNAs, methylations, miRNAs, mRNAs, CAN, mutational expression, or protein level in every sample was presented. The Kaplan–Meier approach having log-rank tests are carried out for comparing global and healthy survival of HCC that have at least a single modification or lack any adjustment within the inquired gene(s). Samples with up-regulation were recognized by the verge of *Z* > 2 (mean expression over 2 SDs). The standard was fixed at 0.05.

### Acquisition of Patient’s Data

LncRNAs appeared as potential features in the field of oncology. RNA-seq data are obtained from TCGA while the exploration of lncRNAs in cancer is provided by an open-access web app “TANRIC.” The TANRIC (The Atlas of ncRNA In Cancer)^2^ allows rapid and intuitive analyses of lncRNAs in the framework of experimental and other molecular information. Through TANRIC, a high amount of lncRNAs were identified with probable biomedical implication, where the majority of them shows robust associations with the already formed therapeutic goals and biomarkers across the cell lines or tumor types. We retrieved lncRNA, miRNA, mRNA, methylation, and protein expression data from TANRIC (Li et al., 2015) of all the TCGA liver cancer patients (Ciriello et al., 2015). The corresponding clinical data are retrieved from Genomic Data Commons (GDC)^3^.

Purity estimation was performed for the patients using consensus purity estimate and the Clonal Heterogeneity Analysis Tool (Li et al., 2012; Li and Li, 2014). Patients were filtered out with purity estimators below 60%.

### Feature Identification for lncRNAs, miRNAs, mRNAs, Methylations, and Proteins

For the identification of promising discriminative lncRNAs, miRNAs, mRNAs, methylations, and proteins of survival groups, the R limma^14^ package was used to identify promising discriminative biomarkers by analyzing the differential expression of lncRNAs, miRNAs, mRNAs, methylations, and proteins.

### Feature Selection of lncRNAs, miRNAs, mRNAs, Methylations, and Proteins

The differentially expressed lncRNAs, miRNAs, mRNAs, methylations, and proteins were used as input features for predictive modeling. Spares learning was applied to select features. The Spares learning and LASSO method were ranked by features based on specific importance.

### Predictive Modeling and Expression Landscape

We used Spares learning and LASSO to construct the predictive model of survival groups. LASSO is a powerful ensemble learning method that has achieved state-of-the-art performance in many biomedical tasks.

(1) $$g_{i}=G_{-I}\,b_{i}+\varepsilon_{i},\mathrm{subject}\,\mathrm{to}\,\left|b_{i}\right|\leq C_{i},$$

where *b*_*i*_ is the coefficient of expressions other than RNA’s *i*, |⋅| is an L-1 norm, and the residual is denoted as ε_*i*_. The *j*_*th*_ coefficient element in *b*_*i*_ indicates a regulatory relationship from RNAs *j* to RNAs *i* (with a direction) in the linear model, where zero shows non-relationship between them. In contrast with correlation-based RNA regulatory networks, linear regression-based RNA regulatory networks can capture the main effects of multiple RNAs. Correlation-based RNA regulatory networks may fail to infer RNA regulation if the correlation is not significantly high and if multiple RNAs regulate simultaneously. The coefficient vector *b*_*i*_ for RNAs *i* is used for constructing the adjacency matrix B of the group-specific RNAS regulatory network, i.e., B = {*b*_1,…,_*b*_*p*_}*T*. Then, Eq. (1) can be optimized by:

(2) $${||g_{i}-G_{-\text{I}}\,b_{i}||}^{2}+\lambda \,\left|{\text{b}}_{i}\right|,$$

where λ is a hyper-parameter for sparsity regularization, and ||⋅||^2^ is an L-2 norm of a vector.

#### Algorithm

1: *I*€{1,…,*p*}**do**

2: ${\text{b}}_{i}^{T}$ = Random LASSO in equation (2)

3: ${\text{b}}_{i}^{C}$ = Random LASSO in equation (2)

4: ***end for***

5: ${\text{B}}^{T}=\left\{{\text{b}}_{I}^{T},\ldots ,{\text{b}}_{p}^{T}\right\}$

6: ${\text{B}}^{C}=\left\{{\text{b}}_{l}^{C},\ldots ,{\text{b}}_{p}^{C}\right\}$

## Results and Discussion

In this study, data from the TCGA Cancer Genomics have been used to explore, visualize, and analyze the genetic and medical features of alterations in cell cycle control found in cases of HCC from databases of TCGA. As per our knowledge, this study is the opening data mining approach that tends to discover the existing connection among modifications occurring in control of cell cycle and patients’ prognosis. A lot of conclusions in this study are coherent with the previously reported data. Remarkably, we detected in our study that alterations in the cell cycle control mostly exist in HCC. Variations in these genetic factors are on autonomous cascades to HCC and are in an uncommon fashion of increasing gene changes. Although no cell cycle control was linked with any of the survival events (disease-free and global survival) in this work, it provides us with a fresh perspective to concurrently investigate biological modifications and medical features through information exploration.

### Genomic Landscape and Alterations Summary

**FIGURE 1:**
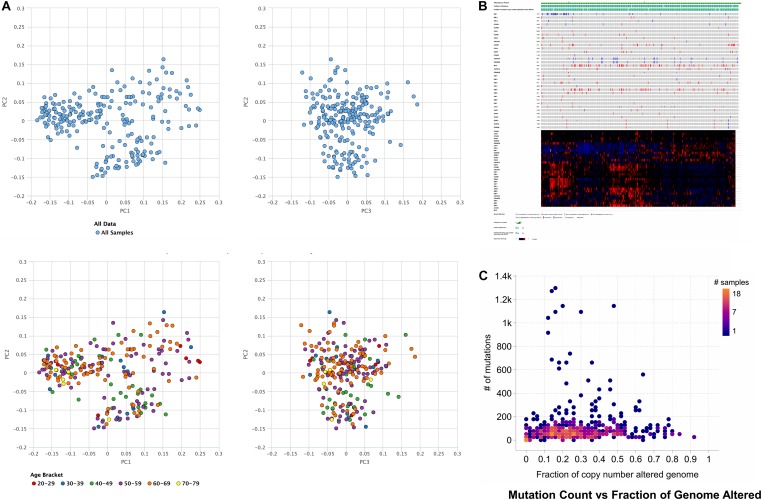
Principal component analysis of hepatocellular carcinoma where the TCGA data and age is **(A)** used for principal component analysis. **(B)** Genetic alterations in the number of samples per patients, illustrating that activation of cell cycle via missense mutations mediates gene signaling in case of the hepatocellular carcinoma, or gene signaling can also be mediated by the inactivation of cell cycle control via truncating mutations. **(C)** The number of mutation counts vs. a fraction of the copy number alterations in the genome.

Based on obtained outcomes, it was observed that the majority of the cases undergo alteration in the cell cycle control, and nearly all of them were missense mutations. Others incorporated deep deletions and few amplifications. However, the rest of the cases remained had modifications in the cell cycle control that comprises most of the truncating and missense mutations. The shared exclusivity analysis implies that events that occurred in cell cycle control were liable to occur again in HCC as shown in Figure 1 through principal component analysis.

### Expression in Cell Cycle Control in HCC

**FIGURE 2:**
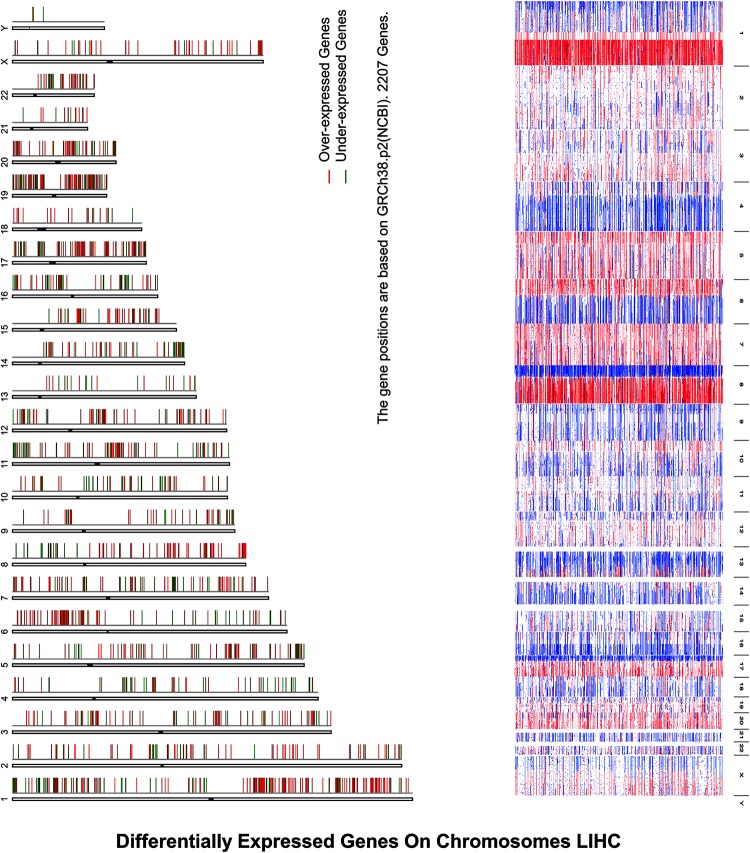
Differentially expressed genes on chromosomes LIHC depict differentially expressed genes on chromosomes in HCC, where expression analysis reveals that there is a significant cell cycle control in under- and overexpressed HCC, explaining that these were hotspots for the activation, where *X*-axis represents the over- and underexpression of genes while *Y*-axis indicates the chromosomes’ number.

Inspection of the expression analysis reveals that there is a significant cell cycle control in under- and overexpressed HCC, explaining that these were hotspots for the activation as illustrated in Figure 2.

### Survival Analysis

**FIGURE 3:**
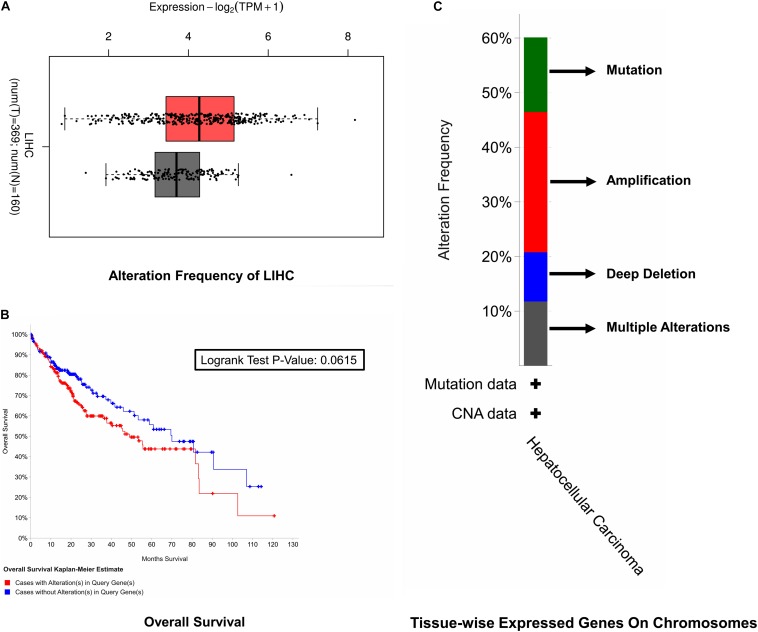
Survival rate inspection. **(A)** Tissue-wise expressed gene on chromosomes. **(B)** Overall survival analysis of the cell cycle control neighborhood in HCC. **(C)** CAN and alteration frequency of cell cycle control in HCC.

For the sake of survival rate inspection, Kaplan–Meier plots were used in an order to complete survival analysis in cases of HCC with as well as without cell cycle control overexpression. For the overall survival analysis, mutations in the cell cycle control were found to be concurrent and not linked to a decreased overall survival (*p* = 0.0615). Likewise, none of the cell cycle control was linked with any of the survival events (Figure 3).

### Liver Cancer Prognosis Markers and Expression Landscape

**FIGURE 4:**
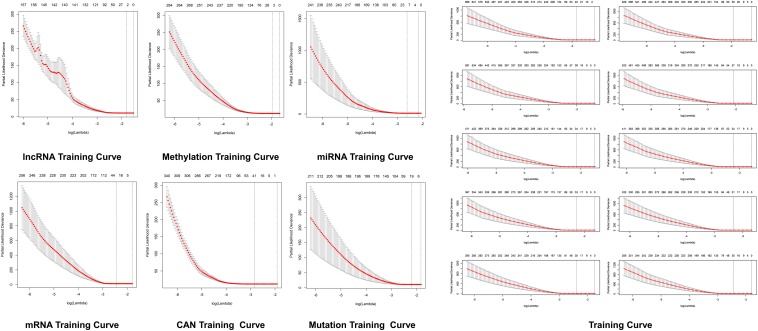
Predictive modeling through LASSO. LASSO with the features weighted training curve in HCC, where optimization equation was resolved through LASSO for RNA implication based on features reputation generation, drilling LASSO with the features biased by reputation, which depend on bootstrapping approach by drilling only a small set of variables instead of drilling the whole variables directly as depicted in figure.

**FIGURE 5:**
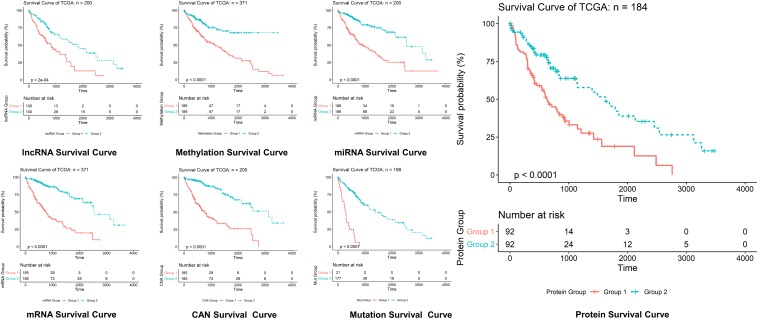
Survival curves prediction through LASSO. Survival curves of lncRNAs, methylations, miRNAs, mRNAs, protein, CAN, and mutation in hepatocellular carcinoma, where optimization equation was resolved through LASSO for RNAs implication based on features reputation generation, drilling LASSO with the features biased by reputation, which depend on bootstrapping approach by drilling only a small set of variables instead of drilling the whole variables directly, and constant approximation is much dependable on every individual training through high-dimensional information as shown here.

It was observed that lncRNAs, miRNAs, mRNAs, methylations, and proteins may exert a more profound biological impact than a single gene by virtue of its intrinsic regulatory nature. Therefore, predictive modeling is also performed for the sake of liver cancer prognosis (Table 1) and the expression landscape of survival patients as shown in Figures 4, 5.

**TABLE 1:** Biomarkers list of lncRNAs, miRNAs, mRNAs, methylations, and proteins in liver cancer with their estimated coefficient score.

**ncRNAs**	**Biomarkers**	**Prediction Scores**
lncRNAs	ENSG00000234577 1	–0.009981348
	ENSG00000236753 1	0.002278461
	ENSG00000257883 1	–0.000943499
	ENSG00000260920 1	0.423349833
	ENSG00000273059 1	–1.47647639
miRNAs	has-mir-139	–0.000141116
	has-mir-148a	−2.26E−07
	has-mir-23c	–0.00822489
	has-mir-3156-1	–0.076147329
	has-mir-3680	0.062526769
	has-mir-3682	0.001235262
	has-mir-3910-2	0.008941494
	has-mir-3924	–0.035235217
	has-mir-548v	0.006351925
	has-mir-592	0.000212203
	has-mir-632	0.107689301
mRNAs	ACSM4	0.212644291
	C15orf33	–0.004560181
	CAD	4.21E−05
	CDCA8	0.000189659
	CHAC2	0.000861851
	DYNC1LI1G6PD	5.19E−05
	G6PD	6.03E−06
	GOT2	−2.16E−06
	GRPEL2	0.000536562
	KLRB1	−8.62E−05
	LDHA	7.57E−07
	MYCN	0.000171789
	MYOD1	0.00620387
	NBPF22P	0.004514996
	PDE6A	0.014750229
	PSD4	−2.48E−05
	PSMD1	352E-05
	RPL21P44	0.00651285
	SEPHS1	0.000165664
	SLC1A7	2.08E−05
	SNAR-C4	0.101384012
	SNORD94	–0.009345186
	TAF3	0.00158546
	TMEM69	3.91E−05
	TTC26	0.000160117
	TXNRD1	1.10E−05
	UCK2	0.000240815
	UTP11L	0.00051883
	YARS	9.13E−06
	ZNF643	1.88E−05
Methylations	Cg02603140	0.0886803
	Cg04104820	–0.612239
	Cg15744128	–0.119538
	Cg24153171	–1.46113
Proteins	Acetyla-Tubulin-Lys40	0.041212138
	B-Raf	0.102643389
	PAI-1	0.114385869

### Predictive Modeling

The optimization of an equation can be resolved by a LASSO explanation. For reliable RNA implication, we applied the Random LASSO (Lee et al., 2014). This technique is divided into two main steps: (1) features’ reputation generation for RNAs and (2) drilling LASSO with the features biased by reputation. This method uses a bootstrapping approach by drilling only a small set of variables instead of drilling the whole variables directly. The constant approximation is much dependable on every individual training through high-dimensional information (Figures 4, 5).

### Validation of Biomarkers

Based on literature reports, an initial major component assessment leads to separation. So, the first thing that we asked was whether the whole organization is practical by using a standardized and shared dataset. Hence, to evaluate our data for equality and applicability, a fivefold leave group cross-validation was employed by the use of LASSO and spare learning. All the datasets were examined distinctly, so each dataset was utilized as a drilling set along with group tags that were equitable to the general receptor position, via reference against liver cancer. Consequently, the receptor status of the entire untrained datasets was predicted by the use of attained Spares learning and LASSO model. Nevertheless, the accuracy of the categorization of the patient’s data builds by the utilization of Spares learning and LASSO models is high. Reasonable Matthews correlation coefficients and low error rates were 0.1 for the grouping of references against liver cancer. The bootstrapping sampling was modified at the time of model formation for addressing inequity in the class in various datasets for drawing an equal number of samples out of each group.

## Conclusion

Detection of cancer at an early curable phase and eradicating the tissues can be capable of preventing the expansion of lethal intrusive cancers, which would save countless lives. Presently, it is extensively stated that lncRNAs, mRNAs, and miRNAs could be probable biomarkers for various cancers. Identification of lncRNAs, mRNAs, and miRNAs related to disease adds to the enhancement of understanding of diagnosis and pathogenesis. Therefore, for the investigation of disease association of lncRNAs, mRNAs, and miRNAs, development of numerous potential computational models has been done. Nevertheless, only some studies centered on the identification of lncRNA, mRNA, and miRNA signs for the diagnosis of liver cancer in the early stage. Consequently, in the current study, we put forward a new classification technique based on lncRNAs, mRNAs, and miRNAs for the categorization of early and advanced phases of liver cancer. The increasing trend in the implementations of machine learning methods and the latest developments of personalized medicine enhanced the forecasting of cancer. For the sake of identifying main aspects that could influence cancer development, recurrence, and survival, different machine learning techniques and algorithms employed for feature selection are globally cast. In general, cancer prediction studies based on machine learning employed expression profiles of mRNA/miRNA, clinical factors, and histological variables as an input for the procedure of cancer prediction. Success in the development of computational models for prediction of cancer rests on comprehending the biological information and shortcomings of the drilling dataset, for instance, minor collection of high-dimensional samples known as “curse of dimensionality.” Nevertheless, the over-drilling problem may be overcome through appropriate feature selection and cross-validation approaches. Our findings provide a new vision for exploring biological functions of lncRNAs, miRNAs, mRNAs, methylations, and proteins in liver cancer, and screening novel potential biomarker (lncRNAs, miRNAs, mRNAs, methylations, and proteins) signature could be a biomarker for the prognosis of liver cancer patients. Better performance toward liver cancer was shown by logistic LASSO regression descriptor where significant improvement was seen in predicting liver cancer.

## Data Availability Statement

The raw data supporting the conclusion of this article will be made available by the authors, without undue reservation, to any qualified researcher.

## Author Contributions

AK, XD, and D-QW designed the experiments. XD, D-QW, and AK performed the entire computational experiments and assisted in writing the manuscript. D-QW and AK analyzed the data and wrote the manuscript. AK, D-QW, XD, and AM read the manuscript and advised on method development. All authors approved the final version of the manuscript.

## Conflict of Interest

The authors declare that the research was conducted in the absence of any commercial or financial relationships that could be construed as a potential conflict of interest.

## Footnotes

1 https://www.cancer.gov/about-nci/organization/ccg/research/structural-genomics/tcga

2 https://ibl.mdanderson.org/tanric/_design/basic/index.html

3 https://gdc.cancer.gov/
